# Endothelial Domes Encapsulate Adherent Neutrophils and Minimize Increases in Vascular Permeability in Paracellular and Transcellular Emigration

**DOI:** 10.1371/journal.pone.0001649

**Published:** 2008-02-20

**Authors:** Mia Phillipson, Jaswinder Kaur, Pina Colarusso, Christie M. Ballantyne, Paul Kubes

**Affiliations:** 1 Department of Medical Cell Biology, Uppsala University, Uppsala, Sweden; 2 Immunology Research Group, Department of Physiology and Biophysics, University of Calgary, Calgary, Alberta, Canada; 3 Center for Cardiovascular Disease Prevention, Methodist DeBakey Heart Center and Baylor College of Medicine, Houston, Texas, United States of America; University of Sheffield, United Kingdom

## Abstract

Local edema, a cardinal sign of inflammation associates closely with neutrophil emigration. Neutrophil emigration has been described to occur primarily through endothelial junctions (paracellular) and more rarely directly through endothelial cells (transcellular). Recently, we reported that unlike in wild-type (wt) mice, Mac-1-/- (CD11b) neutrophils predominantly emigrated transcellularly and was significantly delayed taking 20–30 min longer than the paracellular emigration (wt). In the present study we noted significant anatomical disruption of the endothelium and hypothesized that transcellular emigration would greatly increase vascular permeability. Surprisingly, despite profound disruption of the endothelial barrier as the neutrophils moved through the cells, the changes in vascular permeability during transcellular emigration (Mac-1-/-) were not increased more than in wt mice. Instead increased vascular permeability completely tracked the number of emigrated cells and as such, permeability changes were delayed in Mac-1-/- mice. However, by 60 min neutrophils from both sets of mice were emigrating in large numbers. Electron-microscopy and spinning disk multichannel fluorescence confocal microscopy revealed endothelial docking structures that progressed to dome-like structures completely covering wt and Mac-1-/- neutrophils. These domes completely enveloped the emigrating neutrophils in both wt and Mac-1-/- mice making the mode of emigration underneath these structures extraneous to barrier function. In conclusion, predominantly paracellular versus predominantly transcellular emigration does not affect vascular barrier integrity as endothelial dome-like structures retain barrier function.

## Introduction

A fundamental feature of any inflammatory response is the rapid recruitment of leukocytes from supplying blood vessels to the site of inflammation. The actual emigration out of the vasculature is preceded by leukocyte-endothelial interactions through different adhesion molecules as leukocytes tether to, roll along and adhere on endothelium [1], [2]. The activation of both leukocytes as well as endothelial cells occurs, and is necessary for subsequent emigration [3], [4], [5]. Leukocyte emigration has been described to occur either at endothelial junctions, as leukocytes squeeze between adjacent endothelial cells (paracellular emigration), or directly through endothelial cells (transcellular emigration) (reviewed in 6). Adhesion proteins involved in the emigration process include platelet endothelial cell adhesion molecule-1 (PECAM-1), CD99, ESAM-1, ICAM-1, junctional adhesion molecules and vascular endothelial-cadherins [6], [7]. Although most of these molecules are localized to the junctions, the importance of junctional versus transcellular migration is still debated.

Systematic assessments of leukocyte emigration across endothelium reveal predominant paracellular emigration [8], [9], [10]. This is however challenged by a number of studies showing transcellular emigration of lymphocytes [6], [11], [12] and neutrophils [13], [14] in vitro as well as of neutrophils after exposure to high concentration of bacterial products in vivo [15]. Clearly, the discrepancy in results may simply be due to different stimuli (activating endothelium versus leukocytes), different environmental conditions (eg., different shear), different subsets of leukocytes (eg., lymphocytes versus neutrophils), different organs or even subtleties in the culture conditions used to grow endothelium.

Recently, in identical in vivo conditions, we observed that in a neutrophil response to chemoattractant, a predominant paracellular pathway in wild-type mice versus a predominant transcellular pathway in Mac-1 deficient mice was used [9]. The reason for this difference was related to a new step in the leukocyte recruitment cascade namely neutrophil crawling to junctions, which was absent in Mac-1 deficient mice. Multi-channel fluorescence confocal microscopy revealed that wild-type neutrophils crawled in all directions on the endothelium in order to get to optimal sites for emigration, which for 86% of the neutrophils was at junctions [9]. By contrast, Mac-1-/- neutrophils were unable to crawl to junctional sites for optimal emigration and as a result emigrated primarily at non-junctional sites.

It has been shown that neutrophils bind various adhesion proteins localized to junctional sites to allow for emigration. During this process, a limited amount of protein leaks at these emigration sites, which is referred to as increased permeability. Presumably, more invasive invaginations or penetrations through the endothelium would occur during transcellular migration of neutrophils. Also, the absence of junctional adhesion proteins thought to seal gaps, led us to hypothesis that transcellular emigration would disrupt the barrier to a greater degree further enhancing vascular permeability. Comparing mice where emigration occurs predominantly paracellularly (wild-type mice) versus transcellularly (Mac-1-/- mice), the influence of the route of emigration on endothelial barrier function (microvascular permeability) was investigated. This study shows that the vascular permeability increase was nearly identical in both forms of emigration, mainly as a result of the fact that the endothelium forms a dome over the emigrating neutrophil sealing and thereby limiting changes in vascular permeability independent of the ultimate route of emigration.

## Results

**Figure 1:**
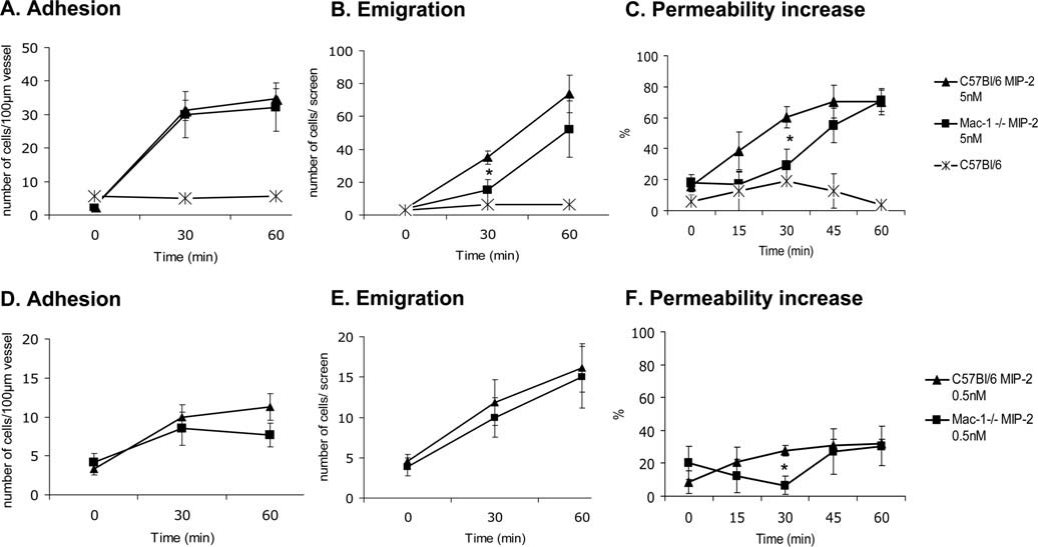
Leukocyte recruitment and vascular permeability, 5 nmol/L MIP-2 (A–C) and 0.5 nmol/L MIP-2 (D–F) superfusion. Leukocyte adhesion (A and D), emigration (B and E) and microvascular permeability increase (C and F) before (time 0) and after addition of MIP-2 (5 nmol/L in A–C and 0.5 nmol/L in D–F) in the superfusate in WT (n = 5 for each concentration) and Mac-1-/- mice (n = 5 for each concentration). Untreated wild-type mice (n = 5) are also included. A leukocyte was considered to be adherent if it remained stationary for more than 30 s, and was quantified as the number of adherent cells within a 100 µm length of venule during 5 min. Leukocyte migration was defined as the number of cells in the extra vascular space within a 200×300 µm area. All values are means±SE. * p<0.05 compared to C57Bl/6 mice receiving similar treatment at the same time-point.

In the first instance we demonstrate that under basal conditions, the cremaster preparation had very little change in leukocyte adhesion (Figure 1A), emigration (Figure 1B) and microvascular permeability (Figure 1C) over 60 min. By contrast, addition of MIP-2, the CXC chemokine that preferentially recruits neutrophils rather than other leukocytes, induced a robust increase in leukocyte recruitment and microvascular permeability at 30 and 60 min. Depletion of neutrophils in our preparations and others essentially eliminated all rolling, adhering and emigrating leukocytes as well as the observed permeability increase clearly demonstrating that the cell type in this model consisted primarily of neutrophils [16], [17]. This was also confirmed using the LyzM-GFP mouse in which only fluorescent cells (myeloid) adhered (data not shown).

Using this model, approximately 70–80 cells min^−1^ rolled in unstimulated blood vessels at a rolling velocity of about 40 µm sec^−1^ in wt mice (data not shown). Mac-1 deficient mice had similar basal values for rolling flux, and slightly higher rolling velocity (about 55 µm sec^−1^). In response to superfusion with the neutrophil specific chemokine, MIP-2, the rolling flux decreased in both groups and was unaffected by the MIP-2 concentration used (0.5 or 5 nmol/L). Rolling cell velocity was not changed over time in response to MIP-2 superfusion in the wild-type mice or the Mac-1-/- mice (data not shown). The number of adherent cells increased about 15-fold in response to MIP-2 (5 nmol/L) in wild-type mice as well as in the Mac-1 deficient mice (Figure 1A). This is in accordance with our previous findings where neutrophil adhesion was dependent on LFA-1 and not Mac-1 [9]. Also emigration was increased in wt mice after 30 min of MIP-2 superfusion, as a 10-fold increase in the number of emigrated cells was observed. After 60 min MIP-2 superfusion, a 20-fold increase was noted (Figure 1B). In the Mac-1-/- mice, despite no effect on adhesion, emigration was significantly delayed. In fact at 30 min there was no significant increase in the number of emigrated neutrophils in response to MIP-2 (Figure 1B). However, at 60 min MIP-2 superfusion, the number of emigrated Mac-1-/- cells was not dissimilar to what was observed in the wild-type mice (Figure 1B). Vascular permeability was measured as the flux of FITC-labeled albumin from the circulation to the interstitial space as we and others have described [18], [19], [20] using computer assisted imaging for quantification. There was a significant increase in permeability over time in response to MIP-2 superfusion in wild-type mice (Figure 1C). It is noteworthy that after 30 min of MIP-2 superfusion, permeability had already increased significantly in wild-type but not in Mac-1-/- mice, which is entirely consistent with the delay in emigration of Mac-1-/- neutrophils. At 60 min, microvascular permeability was increased equally in both strains of mice (Figure 1C). Clearly, the increase in permeability tracked entirely with the number of emigrated, not adherent neutrophils.

It is possible that at 60 min, the maximal permeability increase was achieved in both wt and Mac-1-/- mice, potentially masking any enhancing effects of neutrophil migration through the endothelium (transcellular in Mac-1-/- mice). To address this possibility, we decreased the concentration of MIP-2 10-fold to 0.5 nmol/L. There was significant increase (3-fold less than with 5.0 nmol/L MIP-2) in the number of adherent neutrophils at 30 and 60 min superfusion with the lower MIP-2 concentration in both wild-type mice and Mac-1-/- mice (Figure 1D). At this low concentration of MIP-2, only a 4-fold increase in the number of emigrated cells (25% of high MIP-2) was noted at 60 min (Figure 1E). This resulted in a very subtle increase in vascular permeability with time in both wild-type and Mac-1-/- mice (Figure 1F). Unexpectedly, Mac-1-/- mice did not have enhanced vascular permeability relative to WT mice under these conditions (Figure 1F, n = 5).

**Figure 2:**
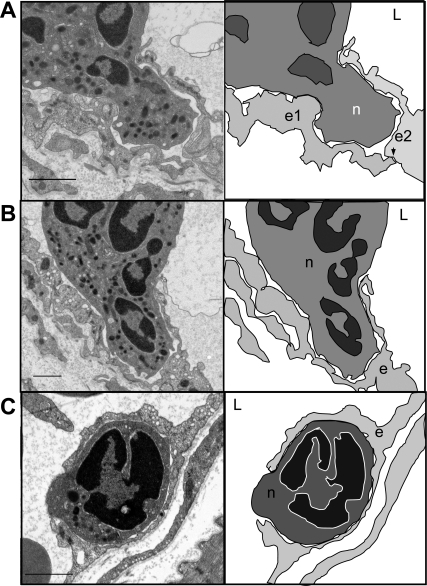
Endothelial transmigratory cup formation. Electron micrographs and cartoons of transmigrating wild-type neutrophils where the endothelium forms a transmigratory cup (A, B and C). The arrowheads mark the junctions and L marks the lumen of the blood vessel. e1, e2 and n represent individual endothelial cells and neutrophils respectively. The bar corresponds to 1 µm.

**Figure 3:**
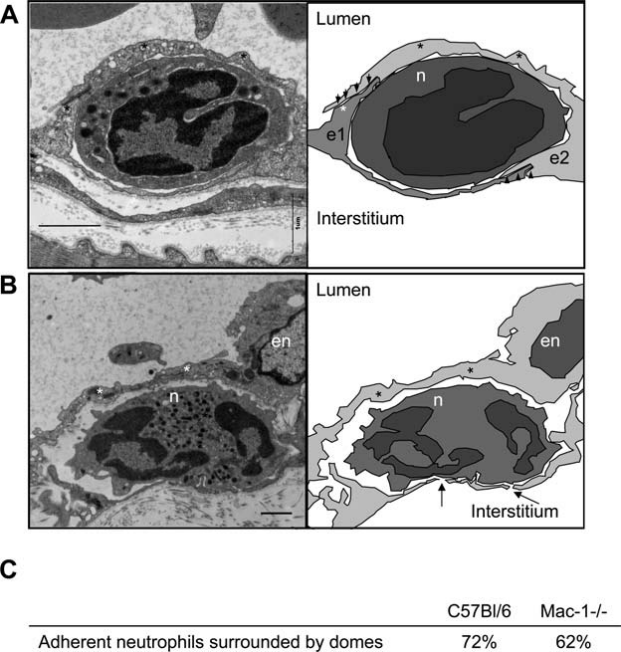
Endothelial enscapsulation of transmigrating neutrophils. Electron micrographs and cartoons of transmigrating wild-type (A) and Mac-1 deficient neutrophils (B). The arrowheads mark the junctions seen in A, the thin endothelial sheet that covers the transmigrating cells is marked with *, e1, e2 and n represent individual endothelial cells and neutrophils respectively. The arrows in B mark gaps in the endothelium, and en marks the endothelial nucleus. Scale bars correspond to 1 µm. The images represent 1 out of 40 analyzed wild-type and 1 out of 24 Mac-1-/- neutrophils. In panel C are presented the percentages of the adherent neutrophils that were covered by endothelial dome-structures.

To further investigate the transmigratory pattern in wild-type versus Mac-1-/- mice, electron micrographs were prepared. In 10% of adherent wild-type and Mac-1-/- neutrophils we identified the endothelium folded into cup-like structures around adherent neutrophils extending a pseudopod (Figure 2A–C). These structures were not dissimilar to what earlier had been described for transmigrating neutrophils in vitro by Carman and Springer [13]. Clearly, similar docking structures occurred in vivo. In addition, we observed in more than 50% of cases that the endothelium extended over the neutrophil creating dome-like structures that completely covered or enveloped the adherent neutrophils (72% of the adherent wild-type neutrophils, Figure 3A and 3C). Continuous endothelium was still seen intact along the basolateral side of the neutrophil. Clearly, the intact endothelium above and beneath the neutrophil, indicates that the endothelium envelopes the neutrophil prior to the leukocyte emigrating out of the vasculature (Figure 3A). These same dome-like structures were also seen in the Mac-1-/- mice (in 62% of the adherent Mac-1-/- neutrophils, Figure 3C) and figure 3B shows a neutrophil covered entirely by a dome and the endothelium underneath the neutrophil beginning to fragment.

**Figure 4:**
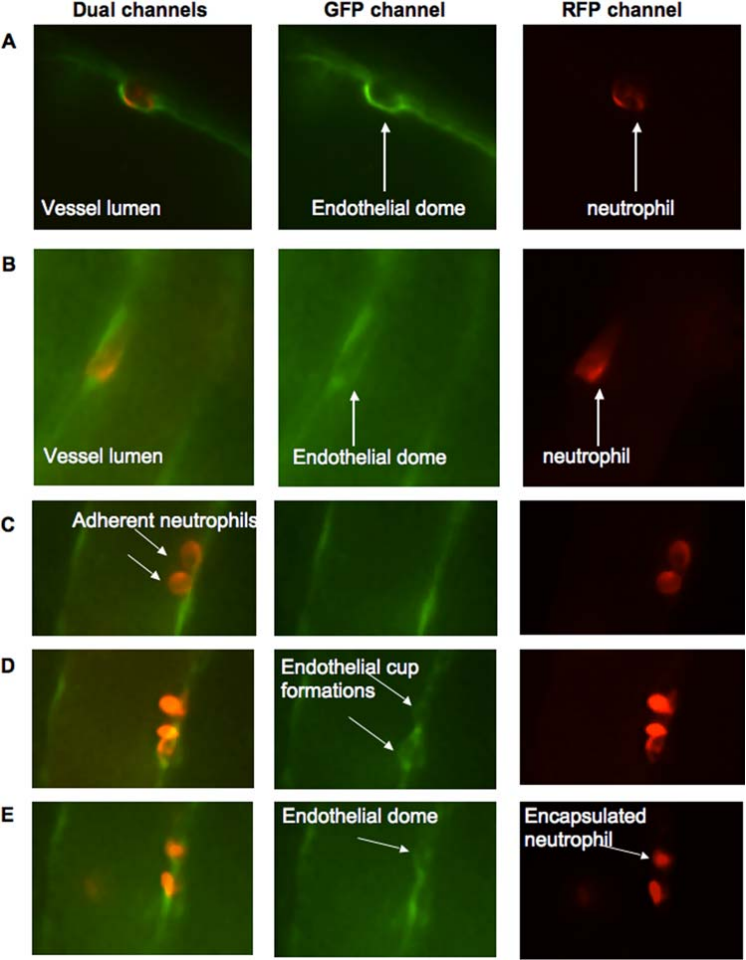
Endothelial dome formation using spinning disk confocal microscopy. Spinning disk confocal images of MIP-2 superfused cremaster muscle of mice with GFP positive endothelium (green) and GR-1 Alexa Fluor 555 stained neutrophils (red). Panels A and B show adherent neutrophils that have been covered by endothelium. By following the same vessel with two adherent neutrophils over time, dynamic formation of endothelial domes was revealed.

It is tempting to conclude from these figures (particularly Figure 2C), that the cup-like structures seen in vitro extended into the dome-like formation of endothelium in vivo. These dome-like structures have not been previously reported in vitro and are presumably not formed or difficult to see in vitro in cell culture conditions. To further explore this possibility, we used spinning disk confocal microscopy in vivo in an attempt to visualize whether domes formed as a result of the movement of endothelium up the sides of the neutrophil thereby enveloping the cell. Figure 4A is a confocal image showing the endothelium (green) completely enveloping the adherent neutrophil (red). In the merged image, a very thin green endothelial dome over the orange neutrophil is visible. Figure 4B shows green endothelium surrounding both sides of the neutrophil. Figure 4C–E shows a series of sequential images where endothelium is moving up the side of two neutrophils and eventually covering one of the neutrophils. It should be emphasized, however, that the endothelial domes were extremely difficult to capture in still images due to their extreme thinness. Nevertheless, Figure 4C–E show the successive steps in the formation of an endothelial dome, from no dome visible to a completely formed dome.

**Figure 5:**
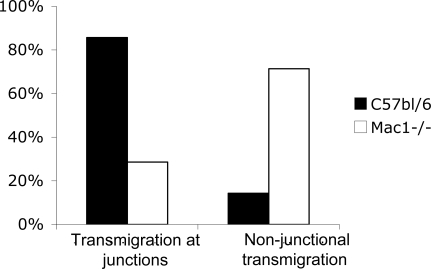
Transmigration in respect to endothelial junctions. The distribution of transmigrating wild-type (n = 40) and Mac-1-/- neutrophils (n = 24) in respect to junctions on electron micrographs.

By using confocal microscopy we have previously reported that wild-type neutrophils emigrated 86% of the time at junctions while Mac-1 deficient neutrophils transmigrated predominantly through the transcellular pathway (61%) [9]. Herein, we examined the electron micrographs of wild-type or Mac-1-/- transmigrating neutrophils and examined the presence or absence of junctions during the transmigration process. This was done by very strict criteria of whether a junction could be seen in the endothelium underneath the adherent neutrophil. Using these parameters, results were very similar to our earlier observation using confocal microscopy [9]. More than 80% of wild-type neutrophils (Figure 5) were transmigrating at a junctional region (as an example see Figure 3A, arrowheads). By contrast 70% of the Mac-1 deficient neutrophils (Figure 5) emigrated with no apparent endothelial junctions seen on the basolateral side of the endothelium (as an example see Figure 3B). Studies of emigrating leukocytes using electron microscopy have been criticized since only a two-dimensional view could be seen leading to the possibility that the junction was simply out of view. Our 3D confocal reconstructions published previously [9] were not subject to this criticism and yet the results were very close to what we observed when 2D electron microscopy was used (Figure 5).

## Discussion

Edema formation is a cardinal sign of inflammation that contributes to the pathogenesis of inflammatory disorders. Leukocytes need to adhere to the endothelial cells in order to induce increased vascular permeability with concomitant tissue swelling and local edema, since anti-CD18 treatment inhibits both leukocyte adhesion and edema development [21]. The current study clearly demonstrates that leukocyte adhesion per se is not responsible for the observed permeability increase, since despite equal number of adherent cells in the wild-type and Mac-1 deficient mice, permeability increased only in the wild-type group at early time points (30 min after MIP-2 addition to the superfusate, Figure 1C). Instead down-stream events from adhesion, namely neutrophil emigration, seem to be responsible for increased vascular permeability. This observation is not dissimilar to studies that showed that chemoattractants given on the luminal side, caused adhesion but no emigration and no vascular permeability increase whereas application of chemoattractants to the ablumenal side increased adhesion, emigration and vascular permeability [22].

Leukocytes can transmigrate out of the vasculature by using either the paracellular or the transcellular route [6]. The factors determining which pathway is preferred are unknown, but the pathway of choice might differ between organs, inflammatory stimuli and the type of leukocyte that is emigrating. Since emigration of neutrophils in vivo seems to occur mainly through the paracellular pathway [9], noting some exceptions including the use of high concentrations of bacterial products [15], one could speculate that transcellular migration is less regulated and might cause greater barrier damage. Recently, using intravital time-lapse video microscopy of muscle microcirculation exposed to MIP-2, we revealed that neutrophils upon adhering to the venular wall crawl to a junction where they then would emigrate [9]. By using a confocal system, it was revealed that in this model 86% of the emigration occurred at junctional regions. We found that adhesion was dependent on the β_2_ integrin LFA-1 (CD11a/CD18), and crawling occurred through activation of the β_2_-integrin Mac-1 (CD11b/CD18) [9]. When the neutrophils were not permitted to crawl due to lack of Mac-1, adhesion was unaffected but 60% of the neutrophils emigrated transcellularly. This served as a model for paracellular (wt) and transcellular migration (Mac-1-/-) under otherwise similar conditions. Surprisingly, we found that the permeability increase of the vascular endothelium was not at all affected by the route of emigration, despite evidence of greater barrier disruption in the Mac-1-/- mice emigrating transcellularly. Electron microscopy however revealed a rate-limiting barrier or dome that sealed the neutrophil from the lumen thereby allowing extensive disruption of the endothelium under the neutrophil without physiologic impact on barrier function. The increase in vascular permeability would then only be a result of the plasma protein that got encapsulated along with the neutrophil.

Ligation of the endothelial adhesion molecules by the adherent leukocytes directly affect the endothelial cells, as demonstrated by transient changes in intracellular calcium, which leads to activation of myosin light chain kinase and endothelial cell shape changes [23]. The induction of endothelial cells to change shape is believed to be important in order for the endothelial cells to retract. This localized dissociation of endothelial cell junctions occurs in order to enable emigration of leukocytes [23]. Newer evidence shows that following firm neutrophil adhesion, the endothelial cells change their morphology so that transmigratory cups are formed, where microvilli-like projections rich in actin, ICAM-1 and VCAM-1 are docking the neutrophil to the endothelium [13]. The endothelial cup-like structures partially engulfing the adherent leukocyte are formed regardless of the emigratory route taken [13]. Electron micrographs also revealed these endothelial docking structures in vivo (Figure 2A) but using both electron microscopy and spinning disk microscopy, we now for the first time show that these docking structures developed into endothelial dome-like encapsulations that completely surrounded the neutrophils (Figure 3 and 4). Interestingly, despite clear evidence that docking structures occur in vitro, dome structures have not previously been observed in vitro suggesting either that the domes do not form in vitro or are simply too difficult to see with presently available technology.

Recently, a new association between endothelial cells and leukocytes was described as transendothelial emigration of lymphocytes was found to be highly dependent on adherent lymphocytes forming podosome protrusions that initiated endothelial invaginations or pseudoprints [24]. Extension of the lymphocyte podosome could eventually lead to pore formation through the endothelial cell, via which the lymphocyte would emigrate [24]. Whether neutrophils could emigrate through transendothelial pores is still unknown, and neutrophil podosome formations have not yet been described in vivo although our electron micrographs did reveal small breaks (potentially pores) below neutrophils (Figure 3B). Contrary to lymphocytes and basophils, neutrophils emigrate in very large numbers during the early immune response. If this occurred through pores, the endothelial encapsulation might be an important phenomenon selectively for neutrophil emigration. Although domes were not described in in vitro studies, it could be that they form in microvascular endothelium under shear conditions when neutrophils are emigrating across an intact vessel wall, conditions difficult to mimic completely in in vitro systems.

Others have previously noted endothelial cytoskeletal rearrangements during neutrophil transmigration after exposure in vivo to either LTB_4_ or fMLP using electron microscopy [15], [25], [26]. This resulted in endothelial domes surrounding transmigrated neutrophils that were suggested to reseal the endothelium after neutrophil diapedesis, but clear gaps were always identified in the endothelium either apically or basolaterally of the neutrophil [25], [26]. In this study, similar but complete encapsulations were observed in the wt and Mac-1 deficient mice, despite the fact that the endothelium on the basolateral side of the Mac-1-/- neutrophils often had multiple gaps directly below the neutrophils (see arrows Figure 3B). However, somewhat unexpectedly the microvascular permeability as an index of barrier function was not higher in the Mac-1-/- than wt group, underscoring the potential importance of these domes. Since the Mac-1-/- neutrophils predominantly emigrate transcellularly, while the wild-type neutrophils exit through junctions, we can from our data conclude that transcellular and junctional emigration seems to cause permeability changes of the same degree. We would suggest that the dome-like endothelial structure completely covering the neutrophil would be the rate limiting structure for vascular permeability, minimizing additional plasma protein leakage regardless of route of emigration.

In conclusion, by comparing mice where emigration occurs predominantly paracellularly versus predominantly transcellularly, this study shows that vascular permeability changes seen during neutrophil recruitment may be limited by an encapsulation of the adherent neutrophil thereby forming an air lock type seal. The permeability would then be independent of the route of emigration. Moreover, the docking structures also referred to as endothelial emigratory cups formed by the endothelium to surround the base of emigrating neutrophils regardless of route of emigration [13] were the initiators of the endothelial domes.

## Materials and Methods

### Animals

Male C57BL/6 mice were obtained from The Jackson Laboratories and Mac-1 deficient male mice were originally made by Dr Ballantyne (Baylor College of Medicine). All mice weighed between 18 and 33 grams. All experimental procedures were approved by the University of Calgary Animal Care Committee and conformed to the guidelines established by the Canadian Council for Animal Care.

### Intravital video-microscopy

The mice were anesthetized with an i.p. injection of a mixture of 10 mg kg^−1^ xylazine (Animal Health; Bayer Inc.) and 200 mg kg^−1^ ketamine hydrochloride (Biomeda-MTC, Animal Health Inc., ON). For all protocols, the right jugular vein was cannulated to administer additional anesthetic. The mouse cremaster muscle preparation was used to study the behavior of leukocytes in the microcirculation and adjacent connective tissue as described previously [27], [28]. In brief, an incision was made in the scrotal skin to expose the left cremaster muscle, which was then carefully dissected free of the associated fascia and continuously kept moist with 37°C bicarbonate-buffered saline. The cremaster muscle was cut longitudinally with a cautery. The testicle and epididymis were separated from the underlying muscle and were gently pushed into the abdominal cavity. The muscle was held flat on an optically clear viewing pedestal and was secured along the edges with five 4.0 sutures. A coverslip with silicon grease on two of the edges was gently placed over the muscle without touching it, to avoid drying of the tissue. An intravital microscope (Optiphot-2; Nikon Inc., Mississauga, ON, Canada) with a ×10 eyepiece and a ×25 objective (Weltzlar L25/0.35; E. Leitz Inc.) was used to examine the microcirculation in the muscle. A video camera (5100 HS; Panasonic) was used to project the images onto a monitor, and the images were recorded for playback analysis using a videocassette recorder.

### Rolling, adhesion and emigration

Single unbranched venules (25–40 µm in diameter) were selected, and to minimize variability, the same section of vessel was observed throughout the experiments. The number of rolling, adherent, and emigrated leukocytes was determined offline during video playback analysis. Rolling leukocytes were defined as those cells moving at a velocity less than that of erythrocytes within the vessel. The flux of rolling cells was measured as the number of rolling leukocytes passing by a given point in the venule per minute. Leukocyte rolling velocity was calculated from the average time required for 10 leukocytes to roll along 100 µm of the vessel. A leukocyte was considered to be adherent if it remained stationary for more than 30 sec, and total leukocyte adhesion was quantified as the number of adherent cells within a 100 µm length of venule over 5 min. Leukocyte emigration was defined as the number of cells in the extra vascular space within a 200×300 µm area (0.06 mm^2^). Only cells adjacent to and clearly outside the vessel under study were counted as emigrated. To induce chemotaxis, the superfusion buffer was changed to MIP-2 superfusion in a concentration of 0.5 or 5 nmol/L for 60 min after the control period.

### Electron microscopy

The cremaster muscle in wt or Mac-1-/- mice was superfused for 60 min with 0.5 nmol/L MIP-2, cut out and processed for transmission electron microscopy. Briefly, samples were fixed with 2.5% glutaraldehyde in 0.1 mol/L cacodylate buffer (pH 7.4) for 2 hours, washed three times with the same buffer, and postfixed with 1% osmium tetroxide in 0.1 mol/L cacodylate buffer (pH 7.4) for 1 hour at room temperature. Then the samples were dehydrated in a graded series of ethanol and embedded in Spurr's resin. Ultrathin sections were obtained with a diamond knife on a ultramicrotome (Ultracut E,, Reichert-Jung, Vienna, Austria), stained with uranyl acetate and lead citrate and then viewed with a Hitachi H-7000 transmission electron microscope (TEMHitachi H-7000, Tokyo, Japan) at 75kV.

### Microvascular permeability measurement

The degree of vascular albumin leakage from cremasteric venules of Mac-1-/- and wt mice was quantified as described previously by both our laboratory [18] and others [19], [20]. In brief, 25 mg kg^−1^ FITC-labeled BSA (Sigma-Aldrich) was administered to the mice i.v. at the start of the experiment, and FITC-derived fluorescence (excitation wavelength, 450–490 nm; emission wavelength, 520 nm) was detected using a silicon-intensified fluorescence camera (model C-2400-80; Hamamatsu Photonics). Image analysis software (ImageJ, the National Institutes of Health, http://rsb.info.nih.gov/ij/), was used to determine the intensity of FITC-albumin-derived fluorescence within the lumen of the venule and in the adjacent perivascular tissue. Background was defined as the fluorescence intensity before FITC-albumin administration. The exposed cremaster muscle was superfused with 0.5 or 5.0 nmol/L MIP-2 in 37°C warmed bicarbonate-buffered saline. The index of vascular albumin leakage (permeability index) at different time points before and after MIP-2 superfusion was determined according to the following ratio expressed as a percentage: (mean interstitial intensity–background)/(venular intensity–background).

### Spinning Disk Confocal Microscopy

The microcirculation of the cremaster muscle was prepared for microscopy as described above and was visualized using a spinning disk confocal microscope. Images were acquired with an Olympus BX51 (Olympus, Center Valley, PA, USA) upright microscope using a 40×/0.75 Plan Fl water immersion objective. The microscope was equipped with a confocal light path (WaveFx, Quorum, Guelph, On. Canada) based on a modified Yokagawa CSU-10 head (Yokagawa Electric Corporation, Tokyo, Japan). Neutrophils were visualized with anti-mouse Ly-6G (Gr-1; BD Biosciences; 2ug/mouse) conjugated with Alexa Fluor 555 (Molecular Probes) injected iv into Tie-2 GFP mice (Background C57Bl/6) to image the neutrophils and endothelium simultaneously. Both 488 and 561 nm laser excitation (Cobalt, Stockholm, Sweden) was used in rapid succession and visualized with the appropriate long pass filters (Semrock, Rochester, New York, USA). Typical exposure times for both excitation wavelengths were 400 ms. A 512×512 pixel back thinned EMCCD camera (C9100, Hamamatsu, Bridgewater, NJ, USA) was used for fluorescence detection. Volocity Acquisition software (Improvision, Lexington, MA, USA) was used to drive the confocal microscope and to remove noise from the captured images. Images captured using the spinning disk were processed and analyzed in Volocity 4.20. The image brightness was adjusted equally in all micrographs to improve visibility.

### Statistics

All data are presented as mean±standard error of the mean (SEM). ANOVA, single-factor, non-repeated measures followed by Fisher protected least significant difference test was performed for multiple comparisons.
